# Cases of Idiopathic Tetanus Successfully Treated by Sedative Remedies

**Published:** 1820-04

**Authors:** 

**Affiliations:** Pavia.


FOR THE LONDON MEDICAL AND PHYSICAL JOURNAL.

### *Cases of Idiopathic Tetanus successfully treated by sedative Remedies.* By Professor Borda, of Pavia. Related by Dr. G. L. Leonardi.

A LABOURING peasant-boy, fourteen years of age, of a weak constitution and ill-developed habit of body, after having been alternately exposed to heat and cold, suddenly became affected with trismus, and afterwards with that universal rigidity of the muscles, which nosologists have called tetanus. He was brought by his parents to the medical ward superintended by Professor Borda. He was soon afterwards seen by the professor, who, supposing the disease to depend on inflammatory affection of the spinal marrow, directed forty leeches to be applied along the vertebral column, a little blood to be taken from the arm, drastic glysters to be injected, and a few grains of tartar emetic to be given in the course of the day dissolved in a small quantity of some liquid.

Notwithstanding these powerful measures, the disease continued its course; so that, on the fifth day, the trismus was almost complete, the opisthotonos very severe, there were violent convulsive affections of the muscles, the pulse was extremely small, and the face had the pallidness of death.

Professor Borda then began to doubt whether he had not exceeded due bounds in the use of the remedies he employed, or whether the disease might not depend on a diathesis different from that which he had suspected. He therefore suspended the use of sedatives, and prescribed an excitant mixture, which was administered with great difficulty, from the trismus, as already stated, being almost complete. He ordered, then, a glyster composed of forty drops of the liquid laudanum of Sydenham in some infusion of chamomile flowers, to be injected every three hours. Matters became still worse under this treatment. The convulsive affections became very frequent, the trismus complete, the tension of the abdominal muscles was so great that they felt like plates of metal, and the opisthotonos increased: the pulse was moderately strong. These results removed all doubts respecting the proper mode of treatment. The use of the excitents was ceased. Emollient glysters were then administered, the tepid bath was used for several hours in continuation, three, and afterwards six, drachms of mercurial ointment were daily rubbed on the skin, from six to eight grains of tartar emetic were administered by the mouth daily, and glysters were injected composed of infusion of chamomile with one drachm of tartar emetic. By these medicines, and the use of blood-letting, the symptoms soon became ameliorated, and the patient in a short time recovered his usual state of health.

Another case of idiopathic tetanus was cured in the same ward by similar means. In this case, the most powerful excitents had been employed in the first stage of the disease under the direction of Dr. Trolli; but, as they were evidently injurious, mercurial frictions, baths, bleeding, drastic glysters, tartar emetic, &c. were afterwards resorted to. The patient soon recovered his health so perfectly, that he shortly afterwards walked from Pavia to Genoa, and returned thence, after a short repose.*

* We have histories of several similar cases, which we shall give on future occasions. Tetanus is of very frequent occurrence in Italy, as most of our readers know; and we hope in a short time to receive thence such a mass of evidence in proof of the benefit of copious blood-letting, tartar emetic, and what Dr. DICKSON of Clifton has so forcibly inculcated the use of, active cathartics, with mercurial frictions in the latter stages of the disease, as to leave no doubt respecting the best method of treating that malady. The histories given above are not so precise and comprehensive as may be desired, and there is a little *coloring* apparently given to them by *sectarism;* but they are highly valuable, notwithstanding the faults here indicated.—EDIT.

